# Study of Myxosporea (Myxozoa), infecting worldwide mullets with description of a new species

**DOI:** 10.1007/s00436-014-4031-5

**Published:** 2014-07-30

**Authors:** V. M. Yurakhno, M. O. Ovcharenko

**Affiliations:** 1 Institute of Biology of the Southern Seas, National Academy of Sciences of Ukraine, 2 Nakhimov Avenue, Sevastopol, 99011 Ukraine; 2 Institute of Biology and Environmental Protection, Pomeranian University, 22b Arciszewskiego Street, 76-200 Słupsk, Poland; 3 Witold Stefański Institute of Parasitology, Polish Academy of Sciences, 51/55 Twarda Street, 00-818 Warsaw, Poland

**Keywords:** Myxozoa, Worldwide mullets, *Myxobolus*, New species

## Abstract

Existing data on Myxozoa parasites infecting mullets were reviewed. The validity of nine species names was updated. Sixteen species were registered during analysis of original material collected in the Mediterranean, Black, Azov, and Japan Seas in 2004–2005. A new bivalvulid myxozoan parasite, *Myxobolus adeli* n. sp., was described from the inner organs of the golden grey mullet *Liza aurata* (Risso, 1810) collected in the Mediterranean (Ebro Delta, Spain), Black Sea (Kerch Strait, Ukraine), and Azov Sea (Genichesk, Ukraine) coastal waters. It is characterized by the presence of elongated, spindle-like cysts 0.5–1.3 mm in size, filled with wide transverse-oval spores about 6.2 × 7.2 × 4.6 μm in size, with two equal polar capsules measuring about 3.0 × 1.8 μm and short polar filament, turned into four coils. The obtained data show that this species differs from all previously described *Myxobolus* spp. with equal polar capsules. Comparative study of *Myxobolus* spp. recorded in worldwide mullets indicates a close relationship with *M. adeli* n. sp. and *Myxobolus improvisus* Isjumova, 1964 registered in mullets. Probably, the last species includes representatives of some different species, infecting freshwater and marine hosts.

## Introduction

The mullets (Mugiliformes: Mugilidae) have a worldwide distribution and inhabit tropical and temperate waters (Nelson 1984). According to current data (FishBase) the Mugilidae family includes 24 genera and 72 species, inhabiting tropical, subtropical, and the southern part of the Atlantic, Indian, and Pacific oceans. Many mullet species have comparatively trivial areas, but one of them—grey mullet *Mugil cephalus* (Linnaeus, 1758)—can be cosmopolitan, spreading including the coastal waters of Europe, Asia, Africa, Australia, America, and Oceania. Mullets have been used as a considerable source of food in different parts of the world. The importance of mullet for aquaculture and the pathologic potential of some parasites, in particular Myxosporea, motivate their detailed study. Myxosporea represents one of the important groups of parasites infecting worldwide mullets (Lom and Dyková 1992; Kent et al. 2001). So far, a few revisionary studies of parasites infecting worldwide mullets have been conducted by Paperna (1975). Twelve species of Myxozoa were reviewed by Paperna and Overstreet (1981). The genera *Sphaerospora*, *Henneguya, Myxidium, Myxosoma*, *Myxobolus, Kudoa*, infecting mullets, were revisionary studied by Sitjà-Bobadilla and Alvarez-Pellitero (1994), Jajasri and Hoffman (1982), Landsberg and Lom (1991), Eiras 2002, Eiras et al. (2005), and Moran et al. (1999).

In the last decades, geography of the mullet parasites studies and knowledge about myxosporeans infecting worldwide mullets were considerably widened. The aim of this paper is to investigate the biodiversity of myxozoans based on existing data and original material obtained during parasitological investigations of mullets in the Mediterranean, Black, Azov, and Japan Seas. Studies were supported by INTAS project (INTAS Ref. No.: 03-51-5998).

## Material and methods

The original study was carried out on data obtained during parasitological investigations of 3,362 fish specimens. Mullets were caught in May–June and October–November 2004–2005. In the Mediterranean coastal region of Spain (Ebro River Estuary and Santa Pola Bay) 1,550 specimens of mullets belonging to five genera were dissected. In the Ponto-Azov region, Ukraine (coastal waters near Kerch, Genichesk, Berdiansk, and Mariupol), 1,498 mullets representing four genera were dissected. Material from the Japan Sea was presented by results of parasitological dissections of 314 mullets from two genera caught in the Russian coastal regions of Japan Sea (Razdolnaya River, Kievka Bay, Posiete Bay, Artemovka). Parasitological analysis was performed based on partial parasitological dissection (Bykhovskaya-Pavlovskaya 1985). Fresh spores were fixed on slides in a glycerine jelly medium according to Donets and Schulman (1973). Spores were photographed and measured on digital images. Descriptions of the spores were based on the references of Schulman et al. (1997) and Lom and Arthur (2006). Live and Giemsa-stained spores were observed and measured under MBI-3 and Olympus BX50F4 microscope equipped with Analysis Pro 2.11 software.

For ultrastructural analyses, infected tissues were fixed in a 2.5 % (*v*/*v*) glutaraldehyde in 0.1 M sodium cacodylate buffer (pH 7.4) for several days at 4 °C. After washing twice with 0.1 M sodium cacodylate buffer and post-fixation in 2.0 % (*v*/*v*) osmium tetroxide in cacodylate buffer for 1 h at 4 °C, the pieces were dehydrated and embedded in Epon–Araldite solution using a standard procedure (Vávra and Maddox 1976). Blocks of embedded tissues were sectioned with an LKB III ultra-microtome. Semi-thin sections were stained with methylene blue. Ultrathin sections were mounted on copper grids, double-stained with uranyl acetate and lead citrate, and examined in a JEM 100B electron microscope operated at 80 kV.

## Results and discussion

### Myxosporeans of the worldwide mullets

By the present time, 64 myxosporean species from 13 genera and nine families infecting 16 mullet species belonging to six genera have been registered (Table 1). Five species were identified to the genus range. The majority of myxosporeans parasitizing mullets are attributed to the family Myxobolidae. Among them, 32 and two species belong to the genera *Myxobolus* and *Henneguya*, correspondingly. Eleven species belong to the family Myxidiidae, eight representatives of *Zschokkella* genus, and three species belong to the genus *Myxidium*. Ten species were found as representatives of the family Kudoidae belonging to a single genus *Kudoa*. The family Sphaerosporidae contains four species belonging to the genus *Sphaerospora*. One species from *Alataspora* and one from *Pseudalataspora* genera were registered as representatives of the Alatasporidae family. Sphaeromyxidae, Ortholineidae, Chloromyxidae, Polysporoplasmidae as well as the Sinuolineidae family are represented by single species of each genus (*Sphaeromyxum*, *Ortholinea, Chloromyxum*, *Polysporoplasma*)*.*

**Table 1:** Myxosporean species infecting mullets

Species of parasite	Species of fish	Site of infection	Localities	Sources
*S. sabrazesi* Laveran & Mesnil, 1900	*L. aurata*, *M. cephalus*	Gall bladder	Black Sea: Sevastopol (Crimea, Ukraine); Mediterranean: Ebro Delta (Spain)	Kolesnikova and Donets (1987), Yurakhno and Ovcharenko (2008), present paper (Fig. 23)
*M. incurvatum* Thélohan, 1892	*M. cephalus*	Gall bladder	Pacific Ocean: California; New Zealand	Jajasri and Hoffman (1982)
*M. leei* Diamant, et al., 1994	Mugilidae gen. sp.	Mucous of the intestine	Marine aquarium: north-east of Spain	Padros et al. (2001)
*M. papernae* Dorothy & Kalavati, 1992	*L. macrolepis*	No data	Indian Ocean	Dorothy and Kalavati (1992)
*Z. admiranda* Yurachno, 1993	*M. cephalus*, *L. aurata*	Gall bladder	Black Sea: Crimea (Ukraine); Mediterranean: Ebro Delta (Spain)	Yurakhno (1993, 2004), Yurakhno and Ovcharenko (2008), present paper. (Fig. 20)
*Z. dogieli* Pogoreltseva, 1964	*M. cephalus*, *L. aurata*, *L. saliens*	Gall bladder	Black Sea: Novorossiysk, (Russia)	Pogoreltceva (1964)
*Z. ganapati* Dorothy *et* Kalavati, 1992	*L. macrolepis*	Gall bladder	Indian Ocean	Dorothy and Kalavati (1992)
*Z. magna* Chen & Hsieh, 1984	*L. haematocheila*	Gall bladder	Liaoho River (China)	Chen and Hsieh (1984)
*Z. mugili* Chen & Hsieh, 1984	*M. cephalus*	Gall bladder	Liaoho River (China)	Chen and Hsieh (1984)
*Z. mugilis* Sitja-Bobadilla & Alvarez-Pellitero, 1993	*L. saliens* (type host), *L. ramada*, *M. cephalus*, *C. labrosus*	Gall bladder	Mediterranean: Ebro Delta (Spain); marine fish farms (Italy)	Sitjà-Bobadilla and Alvarez-Pellitero (1993), Munoz et al. (1999), Quaglio et al. (2002)
*Z. nova* Klŏkačeva, 1914	*M. cephalus*, *L. aurata*, *L. saliens*	Gall bladder	Black Sea: Crimea (Ukraine); Novorossiysk (Russia)	Pogoreltceva (1964), Reshetnikova (1955)
*Zschokkella* sp. Lubat et al., 1989	*L. saliens*	Gall bladder	Adriatic Sea: Boka Kotorska Bay (Montenegro)	Lubat et al. (1989)
*O. divergens* (Thélohan, 1895)	*L. aurata*	Urinary bladder	Black Sea: Sevastopol (Crimea, Ukraine)	Yurakhno (1993, 2004)
*B. indica* Kalavati & Anuradha, 1995	*M. cephalus*	Gall bladder	Backwoods of Visakhapatnam Harbor and Gosthani Estuary, Andhra Pradesh (India)	Kalavati and Anuradha (1995)
*S. corsulae* Sarkar & Ghosh, 1991	*R. corsula*	Gall bladder	Estuary of Hooghly River of Bengal delta near Diamond Harbor, West Bengal (India)	Sarkar and Ghosh (1991)
*S. dicentrarchi* Sitja-Bobadilla & Alvarez-Pellitero, 1992 (Syn. *S. mugili* Yurakhno & Maltsev, 2002; *Sphaerospora s*p. Quaglio et al., 2002; *Sphaerospora* sp. Caffara et al., 2003)	*M. cephalus*, *C. labrosus*, *L. ramada*, *L. aurata*, *L. saliens*	Gall bladder, gut, kidney	Black and Azov Seas: Kerch Strait, Sevastopol, Genichesk (Ukraine); Atlantic ocean; Mediterranean: River Ebro Delta (Spain); marine fish farms (Italy)	Yurakhno and Maltsev (2002), Quaglio et al. (2002), present paper (Fig. 21)
*S. mugili* Asejeva, 2000	*L. haematocheila*	Gall bladder	Razdolnaja River (Russia)	Asejeva (2000)
*S. rostrata* Thélohan, 1895	*Mugil* sp.	Kidney	Mediterranean: coastal waters of Italy and France	Thélohan (1895), Kudo (1919), Sitjà-Bobadilla and Alvarez-Pellitero (1994)
*P. mugilis* Sitja-Bobadilla & Alvarez-Pellitero, 1995	*L. aurata*, *L. ramada*, *Ch. labrosus*	Kidney	Mediterranean: Ebro Delta, Santa Pola (Spain); Black Sea: Sevastopol (Crimea, Ukraine)	Sitjà-Bobadilla and Alvarez-Pellitero (1995), (1996), Yurakhno and Ovcharenko (2008), present paper (Fig. 22)
*Chloromyxum kotorensis* Lubat et al., 1989	*L. aurata*	Kidney	Adriatic Sea: Boka Kotorska Bay (Montenegro)	Lubat et al. (1989)
*Alataspora* sp.	*L. ramada*	Gall bladder	Mediterranean: Ebro Delta (Spain)	Present paper (Figs. 16, 17)
*P. pontica* Kovaljova et al., 1989	*L. aurata*	Gall bladder	Black Sea: Sevastopol (Crimea, Ukraine)	Kovaleva et al. (1989), Yurakhno (1993, 2004)
*M. achmerovi* Schulman, 1966	*M. cephalus*, *L. haematocheila*	Fins, gills, mesentery	Japan Sea: Posiet Bay (Russia)	Schulman (1966), Eiras et al. (2005)
*M. acutus* (Fujita, 1912) Landsberg & Lom, 1991	*M. cephalus*, *L. haematocheila*	Surface of scales	Japan Sea: Peter Great Bay, Tokarjevski Cape; Narva, Kijevka, Avvakumowka, Razdolnaja Rivers (Russia)	Asejeva (1994, 2000)
*M. adeli* sp. n. (syn. *M. improvisus* Isjumova, 1964 (in Schulman 1966 and Yurakhno and Maltsev 2002)	*L. aurata*	Intestine, swim bladder, pyloric caeca, esophagus, stomach, gills	Black and Azov Seas: Kerch Strait, Genichesk, Sevastopol (Crimea, Ukraine); Mediterranean: Ebro Delta, Santa Pola (Spain)	Yurakhno and Maltsev (2002), present paper (Figs. 2, 3, 28)
*M. anili* Sarkar, 1989	*R. corsula*	Mesentery associated with duodenum	Indian Ocean: Bay of Bengal (India)	Sarkar (1989)
*M. bankimi* Sarkar, 1999	*S. cascasia*	Gall bladder	Parganas, West Bengal (India)	Sarkar (1999)
*M. bizerti* Bahri & Marques 1996 (syn. *M. hannensis* Fall et al., 1997)	*M. cephalus*	Gills	Mediterranean: Ichkeul, Bizerte, Ghar El Melh; Atlantic Ocean: Baje de Gorée (Senegal)	Bahri and Marques (1996), Eiras et al. (2005), Fall et al. (1997), Bahri et al. (2003), Yemmen et al. (2012)
*M. bramae* Reuss, 1906	*M. cephalus*	Gills, gill arches, skin, fins, muscles, mouth, esophagus, intestine, gall bladder, swim bladder, kidney, liver, spleen, heart	Azov and Black Seas: Kerch Straite (Crimea, Ukraine)	Iskov (1989), Yurakhno and Maltsev (2002)
*M. branchialis* (Markevitsch, 1932) Landsberg & Lom, 1991	*M. cephalus*, *L. aurata*, *L. saliens*	Gill filaments, kidney, and spleen	Black and Caspian Seas	Schulman (1966), Ibragimov (1987), Iskov (1989)
*M. cephalis* Iversen et al., 1971	*M. cephalus*	Braine meninges, gill arches, buccal cavity, jaw bone, crop tissue	Atlantic Ocean: Mexical Gulf (USA)	Iversen et al. (1971), Lom and Dyková (1992), Eiras et al. (2005)
*M. cheni* Schulman, 1962	*M. cephalus*, *L. haematocheila*	Trunk muscles	Liaoho River (China)	Schulman (1962, 1966), Eiras et al. (2005)
*M. circulus* (Achmerov, 1960)	*M. cephalus*	Gills, muscles, kidney, fins, separate spores in other organs	Black Sea: Paleostomi Lake (Georgia); Lyubimovka (Crimea, Ukraine)	Naidenova et al. (1975), Iskov (1989), Yurakhno (2004)
*M. episquamalis* Egusa et al., 1990	*M. cephalus*	Beneath the scales, fins, gill arches	Mediterranean: Ichkeul lagoon (Bizerte, Tunisia); coastal waters of Japan and Korea; estuaries in eastern Australia; Mediterranean: Camlik lagoon (Turkey); Santa Pola (Spain); Atlantic Ocean: Senegalese coast	Egusa et al. (1990), Eiras et al. (2005), Lom and Dyková (1994), Bahri and Marques (1996), Rothwell et al. (1997), Bahri et al. (2003), Yurakhno and Ovcharenko (2008), Özak et al. (2012), Diamanka et al. (2008), Kim et al. (2013), present paper (Figs. 4, 7)
*M. exiguus* Thélohan, 1895	*M. cephalus*, *C. labrosus*, *L. aurata*, *L. saliens*, *L. ramada*	Gill filaments, gill arches, pyloric caeca, heart muscles, stomach cavity, gall bladder, intestine, kidney, mesentery, spleen, fins	Mediterranean: Marsel, Banyuls (France); Genuya, Napoli (Italy); Adriatic Sea: Boka Kotorska Bay (Montenegro); Tunisian lagoons; Narva and Kijevka Rivers (Russia); Caspian Sea (Middle and southern parts of Turkmenian Gulf; Azov and Black Seas (Ukraine); Atlantic ocean (France), Baie de Goree (Senegal)	Thélohan (1895), Parisi (1912), Kudo (1919), Schulman (1957, 1966), Ergens et al. (1975), Siau (1978), Pulsford and Matthews (1982), Iskov (1989), Lubat et al. (1989), Lom and Dyková (1992), Fall et al. (1997), Asejeva (2000), Eiras et al. (2005), present paper
*M. goensis* Eiras & D’Souza, 2004	*M. cephalus*	Gills	Coast of India	Eiras and D’Souza (2004), Eiras et al. (2005)
*M. ichkeulensis* Bahri & Marques, 1996 (syn. *M. goreensis* Fall et al., 1997)	*M. cephalus*	Gills, muscles, skin, scales	Mediterranean: Ichkeul lagoon (Bizerte, Tunisia); Lake Ichkeul (Tunisia), Camlik lagoon (Turkey); Santa Pola, Ebro Delta (Spain); Black and Azov Seas: Kerch Strait, Genichesk (Crimea, Ukraine); Atlantic Ocean: Baje de Gorée (Senegal)	Bahri and Marques (1996), Fall et al. (1997), Bahri et al. (2003), Eiras et al. (2005), Pedro-Andrĕs et al. (2011), Özak et al. (2012), present paper (Figs. 5, 6)
*M. lizae* (Narasimhamurti & Kalavati, 1979) Landsberg & Lom, 1991	*L. macrolepis*	Outer wall of the gut	Indian waters at Andhra Pradesh (India)	Narasimhamurti and Kalavati (1979a), Eiras et al. (2005)
*M. muelleri* Bütschli, 1882	*M. cephalus*, *L. aurata*, *L. saliens, L. ramada*	Gills, mesentery, intestine, gall and urinary bladders, liver, kidney, gonads, spleen, eyes, fins, heart, muscles	Mediterranean: Napoli (Italy), Ichkeul lake (Tunisia); Azov and Black Seas: Evpatoriya, Karadag, Sevastopol, Kerch Strait, Genichesk (Crimea, Ukraine); Atlantic Ocean: Bai de Goree (Senegal)	Parisi (1912), Pogoreltceva (1952, 1964), Reshetnikova (1955), Bahri et al. (2003), Eiras et al. (2005), present paper (Fig. 1)
*M. mugauratus* (Pogoreltceva, 1964) Landsberg & Lom, 1991	*L. aurata*	Mesentery	Black Sea: Sudak (Crimea, Ukraine)	Pogoreltceva (1964)
*M. mugcephalus* (Narasimhamurti et al., 1980) Langsberg & Lom, 1991	*M. cephalus*	Gill filaments	Indian coastal waters	Narasimhamurti et al. (1980), Eiras et al. (2005)
*M. mugchelo* (Parenzan, 1966) Landsberg & Lom, 1991	*C. labrosus*	Gills	Mediterranean: Gulf of Tarento	Parenzan (1966), Eiras et al. (2005)
*M. mugilis* Perugia, 1891	*L. aurata*, *L. ramada*	No data	Mediterranean	Perugia (1891)
*M. mugilii* Haldar et al., 1996	*M. cephalus*	No data	Indian Ocean: Bay of Bengal, Orissa (India)	Haldar et al. (1996), Eiras et al. (2005)
*M. narassii* (Narasimhamurti, 1970) Landsberg & Lom, 1991	*L. vaigiensis*	Gut epithelium	Indian coastal waters	Narasimhamurti (1970), Eiras et al. (2005)
*M. nile* (Negm-Eldim et al., 2005	*M. cephalus*	Gills	Egypt; Mediterranean: Ebro Delta (Spain)	Negm-Eldim et al. (1999), Eiras et al. (2005), present paper (Fig. 14)
*M. parenzani* (Parenzan 1966) Landsberg & Lom, 1991	*C. labrosus*	Gills	Mediterranean: Gulf of Tarento (Italy)	Parenzan (1966), Eiras et al. (2005)
*M. parvus* Schulman, 1962	*M. cephalus*, *L. haematocheila*	Gill lamellae, gall bladder, kidney, intestine, liver, mesentery	Liaoho River (Chine), Japan Sea; Azov Sea; Black Sea	Schulman (1962), Karatajev and Iskov (1984), Domnich and Sarabeev (1999, 2000), Sarabeev and Domnich (2000), Syirovatka and Nizova (2000), Eiras et al. (2005), present paper (Figs. 8–13)
*M. platanus* Eiras et al., 2007	*M. platanus*	Spleen	Lagoa dos Patos (Brasil)	Eiras et al. (2007)
*M. raibauti* Fall et al., 1997	*M. cephalus*	Liver	Atlantic Ocean: Baje de Gorée (Senegal)	Fall et al. (1997), Eiras et al. (2005)
*M. rohdei* Lom & Dykova, 1994	*M. cephalus*	Kidney, gall bladder, intestine, mesentery, muscles	Estuary of Arrawarra creek (Australia); Mediterranean: Delta Ebro (Spain)	Lom and Dyková (1994), Eiras et al. (2005), present paper (Fig. 19)
*M. rotundus* Nemeczek, 1911	*L. aurata*	Gill lamellae; heart and other inner organs	Black Sea: Paleostomi Lake (Georgia)	Donets (1979), Iskov (1989)
*M. spinacurvatura* Maeno et al., 1990	*M. cephalus*	Intestine, liver, intrahepatic bile ducts and gall bladder, spleen, mesentery, mesenteric vessels, brain, liver, spleen, pancreas, gill filaments	Mediterranean: Ichkeul lagoon, Bizerte (Tunisia), Lake Ichkeul in northeastern Tunisia, Delta Ebro, Santa Pola (Spain); Narva River (Russia), Estuary of Arrawarra creek, New South Wales coast (Australia), Japan coastal waters	Maeno et al. (1990), Lom and Dyková (1994), Bahri and Marques (1996), Asejeva (2000), Bahri et al. (2003), Eiras et al. (2005), present paper (Fig. 15)
*M. supamattayai* Kittichon et al., 2011	*V. seheli*	Skin	Andaman Sea (Thailand)	U-Taynapun et al. (2011)
*Myxobolus* sp. Faye et al., 1997	*M. curema*	Heart	Atlantic coast of Senegal	Faye et al. (1997)
*Myxobolus* sp. Yemmen et al., 2012	*M. cephalus*	Liver	Mediterranean: Ghar El Melh lagoon (Tunisia)	Yemmen et al. (2012)
*Myxobolus* sp. II. Yemmen et al., 2012	*M. cephalus*	Heart	Mediterranean: Ghar El Melh lagoon (Tunisia)	Yemmen et al. (2012)
*H. ouakamensis* Kpatcha et al., 1997	*M. cephalus*	Heart, gills	Atlantic coast (Senegal)	Kpatcha et al. (1997), Eiras (2002)
*Henneguya* sp. Faye et al., 1997	*M. cephalus*	Heart	Atlantic coast (Senegal)	Faye et al. (1997)
*K. bora* (Fujita, 1930)	*M. cephalus*, *M. japonica*, *L. carinata*	Musculature	Pacific Ocean (Taiwan)	Fujita (1930)
*K. cascasia* Sarkar & Chaudry, 1996	*S. cascasia*	Mesentery associated with intestine	Indian Ocean (Bay of Bengal)	Sarkar and Chaudhury (1996)
*K. haridasae* Sarkar & Ghosh,1991	*L. parsia*	Gall bladder	Estuarine waters of West Bengal (India)	Sarkar and Ghosh (1991)
*K. intestinalis* Maeno et al., 1993	*M. cephalus*	Intestinal musculature	Southeastern coast of the Kii Peninsula (Gokasho Bay, Japan)	Maeno et al. (1993)
*K. iwatai* Egusa & Shiomitsu, 1983	*M. cephalus*	Muscles, adipose tissue, nerve axons, mesentery, swim bladder, heart, pericardium, kidney, ovary	Red Sea farms in Gulf of Eilat (Israel)	Diamant et al. (2005)
*K. quadratum* (Thélohan, 1895)	*M. cephalus*	Musculature	Black Sea: Karadag, Sevastopol (Crimea, Ukraine)	Iskov (1989)
*K. tetraspora* Narasimhamurti & Kalavati, 1979	*M. cephalus*	Braine, optic lobes	Indian Ocean: coast of India	Narasimhamurti and Kalavati (1979b)
*K. trifolia* Holzer et al., 2006	*L. aurata*, *L. ramada*	Connective tissue of spleen, kidney, gall bladder, swim bladder, intestine, intestinal mesentery, gills	Mediterranean: Santa Pola (Spain)	Holzer, et al. (2006), present paper (Figs. 18, 25)
*K. unicapsula* Yurakhno et al., 2007	*L. ramada*, *L. aurata*	Intestinal mesentery, pyloric caeca	Mediterranean: Santa Pola, Ebro Delta (Spain)	Yurakhno et al. (2007), present paper (Figs. 24–27)
*K. valamugili* Kalavati & Anuradha, 1993	*V. cunnesius*	Intestinal musculature	Indian Ocean: Visakhaptnam harbor (India)	Kalavati and Anuradha (1993)

The maximum of species richness of Myxosporea was registered in flathead mullet *M. cephalus*. Thirty six species of myxosporeans from eight genera were mentioned in named host. The area includes the Mediterranean basin, Red Sea, Atlantic Coast of Africa, Mexican Gulf, and Indian and Pacific Ocean coastal waters.

Golden grey mullet *Liza aurata* (Risso, 1810) was mentioned as the host of 18 species of Myxosporea infecting different organs of the host in the Mediterranean, Black, and Azov Seas. Leaping mullet *Liza saliens* (Risso, 1810) is a host of nine species of Myxozoa, found in the Black, Azov, Mediterranean, Adriatic, and Caspian Seas. Nine species of myxosporeans were also found in thinlip mullet *Liza ramada* (Risso, 1810) from the Mediterranean basin. Six species of Myxosporea were described in thicklip grey mullet *Chelon labrosus* (Risso, 1827) and in redlip mullet *Liza haematocheila* Temminck & Schlegel, 1845 in the Japan Sea (Russia), in Liaohe River (China), and in Black and Azov Seas (Ukraina). From the Indian shores, three species of myxosporeans were found in largescale mullet *Liza macrolepis* (Smith, 1846) and two species in corsula *Rhinomugil corsula* (Hamilton, 1822) and in yellowtail mullet *Sicamugil cascasia* (Hamilton, 1822). One species was described from squaretail mullet *Liza vaigiensis* (Quoy & Gaimard, 1825), *Liza parsia* (Hamilton, 1822), and longarm mullet *Valamugil cunnesius* (Valenciennes, 1836), from *Mugil japonica* and keeled mullet *Liza carinata* (Valenciennes, 1836), white mullet *Mugil curema* Valenciennes, 1836 and *Mugil platanus*.

Among the species of myxosporeans, described from mullets, 17 species were found in the gall bladder. In the gills, muscles, and kidney, consequently, six, five, and four species of myxosporeans were registered. Three myxozoans species were found in the mesenterium and intestines; two in the heart, on fins, and scales. The urinary bladder, spleen, and liver were infected with a separate species of myxozoans. Eighteen species were detected in various organs (Table 1).

There are only six cosmopolite species. All of them are parasites of *M. cephalus*. Those are *Myxobolus muelleri, Myxobolus ichkeulensis*, *Myxobolus episquamalis*, *Myxobolus exiguus*, *Myxobolus parvus*, *and Myxobolus spinacurvatura*.

### Original data of the author’s investigations

We conducted taxonomical studies of mullet myxosporeans collected in the Mediterranean, Black, Azov, and Japan Seas in the summer and autumn 2004–2005. *M. cephalus* was parasitologically studied in all regions; *L. haematocheila—*in the Japan, Black, and Azov Seas; *L. aurata* and *L. saliens—*in the Mediterranean, Black, and Azov Seas; and *L. ramada* and *C. labrosus*—exclusively in the Mediterranean Sea.

Totally, 16 species of myxosporeans have been registered. New information about myxosporean fauna for each region of investigations has been received.

*Zschokkella admiranda* from *M. cephalus* has been registered for the first time in the Mediterranean fauna. *Sphaeromyxa sabrazesi*, *Kudoa unicapsula*, *Alataspora* sp., *Z. admiranda*, *Myxobolus adeli* sp. n*.*, *M. parvus*, *M. muelleri*, *M. ichkeulensis*, *M. spinacurvatura*, *Myxobolus rohdei*, *M. exiguus*, *Myxobolus nile*, *Myxobolus episquamalus* have been found in the coastal waters of Spain*. M. cephalus* appeared to be a new host for *S. sabrazesi.* *L. aurata* was registered as a new host for *Sphaerospora dicentrarchi. L. ramada* and *C. labrosus* were found as hosts for *Polysporoplasma mugilis* in the Mediterranean Sea. *P. mugilis* infecting *L. aurata* has been found for the first time in the Black Sea. *S. dicentrarchi*, *M. ichkeulensis,* and *M. spinacurvatura* infecting *M. cephalus* was firstly registered in the Black and Azov Seas. *L. aurata* was firstly registered as a new host for *Z. admiranda. M. ichkeulensis, M. spinacurvatura*, and *M. episquamalus* parasitizing *M. cephalus* has been found for the first time in the Japan Sea.

Among mullets inhabiting the Mediterranean basin, we found several myxosporeans, already known species of parasites, which were described earlier as new species. All of them were synonymized. Species names *Sphaerospora mugili* Yurakhno & Maltsev, 2002; *Sphaerospora* sp. Quaglio et al., 2002; and *Sphaerospora* sp. Caffara et al., 2003 were considered as younger synonyms of *S. dicentrarchi* Sitja-Bobadilla & Alvarez-Pellitero, 1992. Others species names containing synonyms are presented by as follows: *Myxobolus bizerti* Bahri & Marques, 1996 (=*Myxobolus hannensis* Fall et al., 1997); *Myxobolus ichkeulensis* Bahri & Marques, 1996 (=*Myxobolus goreensis* Fall et al., 1997), *M. adeli* sp. n. (=*Myxobolus improvisus* Isjumova, 1964 (in Schulman 1966; Yurakhno and Maltsev 2002); *Myxobolus lizauratus* (in Yurakhno and Ovcharenko 2008).

In the present paper, we describe the following new species: *M. adeli* sp. n. from *L. aurata* in the Mediterranean, Black, and Azov Seas.

*Myxobolus adeli* sp. nov. (Table 2; Figs. 2, 3, 28)

**Table 2:** Comparative data of *Myxobolus adeli* sp. n. and three closely related *Myxobolus* spp.

Species	*Myxobolus adeli* sp. n.	*Myxobolus improvisus*	*Myxobolus latus*	*Myxobolus artus*
Shape and sizes of vegetative plasmodia	Spindle-form, 0.5–1.3 mm	Round 1.5 mm, in diameter	Round, not more than 0.5 mm in diameter	Round or oval, not more than 0.5 mm in diameter
Spore length (μm)	5.56–6.75	6.5–7.7	7.0–10.0	6.5–8.5
Spore width (μm)	6.57–7.77	7.5–9.3	8.4–11.0	9.0–12.0
Spore thickness (μm)	3.55–5.27	–	5.2–5.6	5.5
Polar capsule length (μm)	2.36–3.8	4.6–5.6 and 3.7–4.0	4.0–5.6	4.0–6.0
Polar capsule width (μm)	1.26–2.28	2.0–3.3 and 2.6	3.0–4.0	2.3–5.0

**Figs. 1–28:**
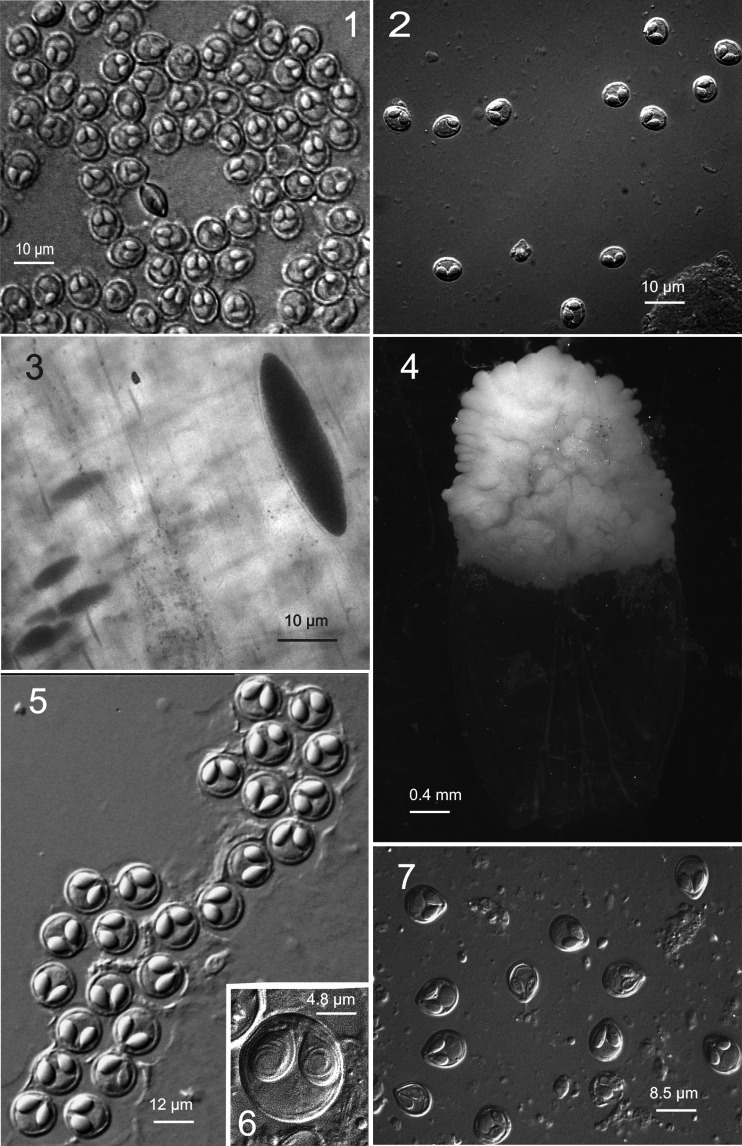

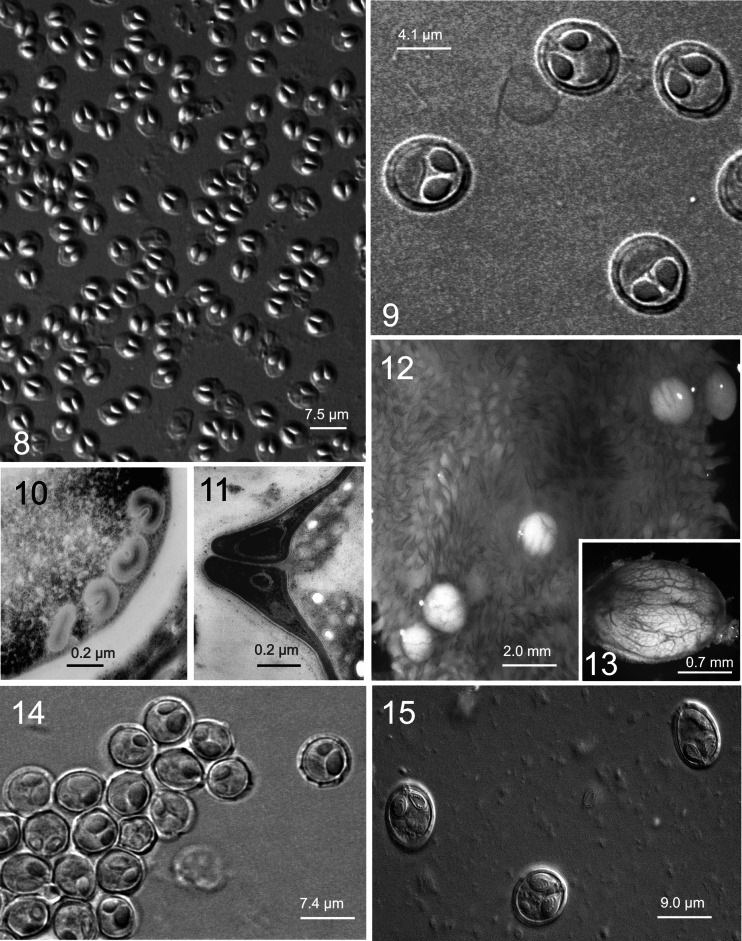

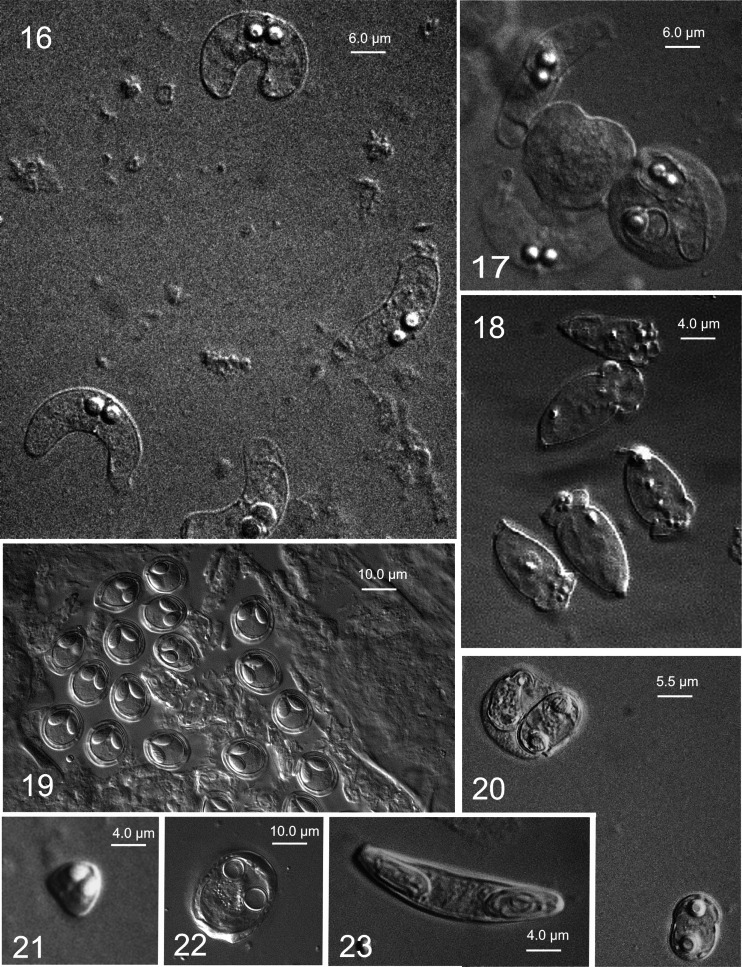

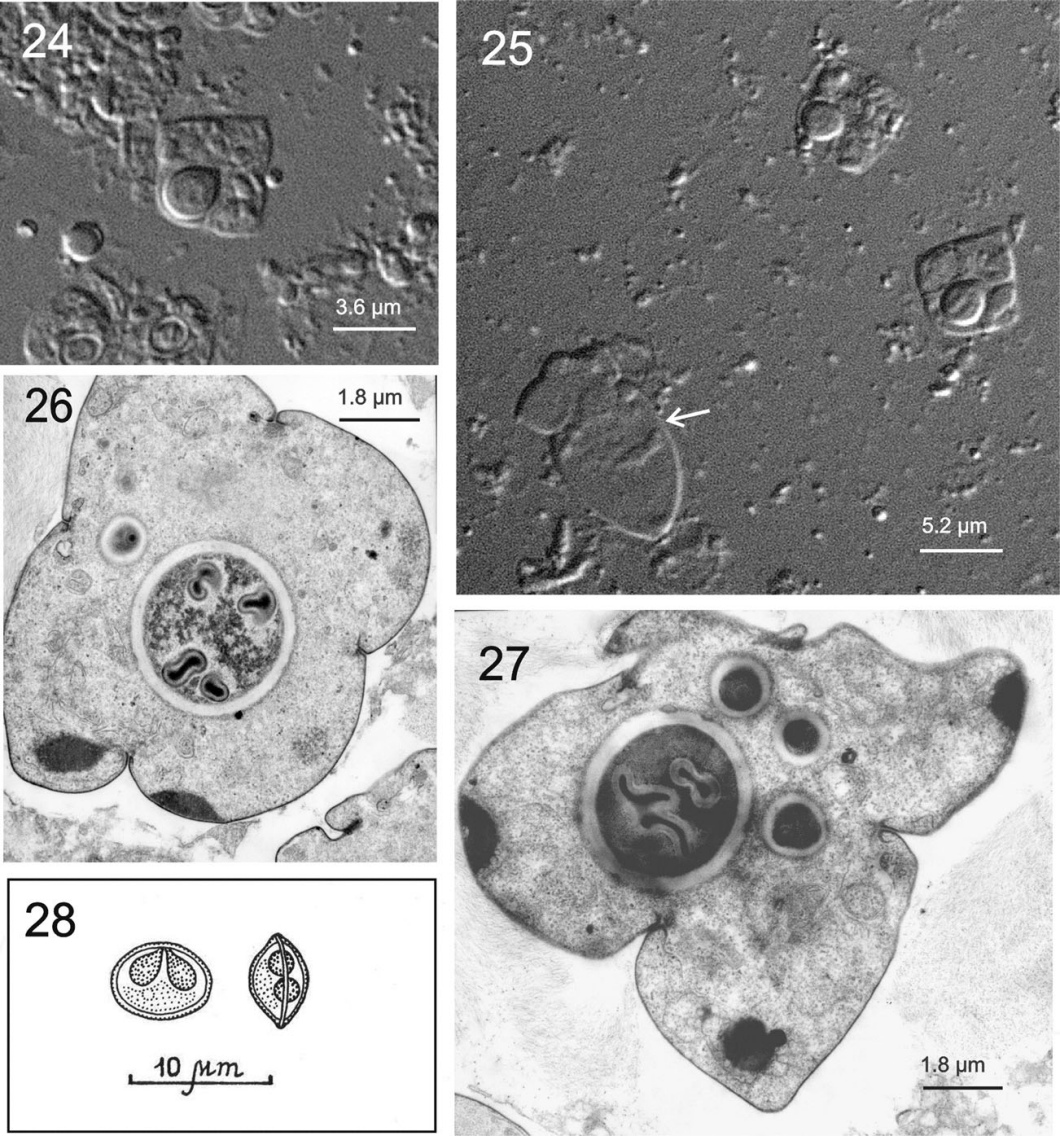
Light microscope and ultrastructural data of some myxozoan parasitizing collected mullets. *1* Spores of *M. muelleri. 2*, *3 M. adeli* sp. nov., spores (*2*) and spindle-shaped cysts of different maturity (*3*). *4*, *7 M. episquamalis.* Compact whitish masses on the distal parts of scales (*4*). Each cystic mass consists of numerous microcysts. Oval spores tapered at the anterior end (*7*). Polar capsules equal and pyriform. *5*, *6* Spherical spores of *M. ichkeulensis* with oval polar capsules. No intercapsular appendix is visible (*6*). *8–13 M. parvus.* Spores (*8–11*) and rounded-to-oval white cysts up to 2.0 mm in diameter (*12*, *13*). Polar capsules contain four coils of longitudinally twisted polar filament (*10*). Two valvogenic cells form a good developed sutural ring (*11*). *14* Spores of *M. nile* with unequal polar capsules. *15* Spores of *M. spinacurvatura.* Polar capsules do not reach the midpoint of the spore length. *16–17 Alataspora* sp*.* Spherical polar capsules located close to the anterior pole (*16*). Vegetative stages presented by rounded or oval-shaped bisporous plasmodia with transparent ectoplasm and small-grained endoplasm (*17*). *18 Kudoa trifolia.* Four small subspherical polar capsules are located in the central part of the spore, between the spore body and leaf-like appendages. *19 M. rohdei.* Spores are regularly ellipsoidal with a good developed sutural edge around the spore, bearing distinct sutural markings. *20 Z. admiranda.* Round or oval disporous plasmodia with small granular endoplasm. Oval spores with rounded poles. *21* Spores of *S. dicentrarchi. 22 P. mugilis.* Spores subspherical in front view. Sutural line straight. Polar capsules spherical, of equal size. *23 S. sabrazesi.* Spores cylindrical, bent in arch form; with truncated ends. Polar capsules large, cylindrical. *24–27* Light and electron microscope data of the spores of *K. unicapsula. K. unicapsula* and *K. trifolia—*mix infection (*25*). *26–27* Ultrastructure of the spores of *K. unicapsula* (*26*, *27*). Transverse (*26*) and cross (*27*) sections through the basal part of the spore showing unequal polar capsules and four shelves. Big polar capsule contains two coils of polar filament. *28—*Spore construction of *M. adeli* sp. nov. Host infected: *M. cephalus* (*4–7*, *14*, *15*, *19*, *21*, *23*); *L. aurata* (*1*, *8–13*, *20*, *22*, *24–27*); *L. ramada* (*2*, *3*, *16–18*, *28*). Sites: intestine (*1*, *8–13*, *15*, *19*, *24–27*), pyloric caeca (*2*, *3*), scales (*4*, *7*), gills (*14*), gall bladder (*16*, *18*, *20*, *21*, *23*), and kidney (*22*)

*Type host*. Golden mullet *L. aurata* (Risso, 1810)

*Site of infection*. Intestine, pyloric caeca, esophagus, stomach, swim bladder; sporadically: gills and muscles

*Locality.* Mediterranean coastal waters (Ebro River Delta, Santa Pola Bay), Black Sea waters (Kerch Channel), and Azov Sea (Genichesk aquatoria)

*Prevalence*. Ebro River Delta, Spain, autumn 2005—11 % (8/73); Santa-Pola Bay, Spain, summer 2005—12 % (7/60); Kerch Channel, Ukraine, summer 2004—13 % (11/83), autumn 2005—11 % (4/35); Genichesk, Ukraine, summer 2004—6 % (11/188), autumn 2005—9 % (4/47)

*Description*. Vegetative forms: cysts are spindle form with sharpened or rounded ends, 0.5–1.3 mm in size. Spores: oval shaped, transversally widened. Widely positioned pyriform polar capsules close acquired at the anterior pole and occupy half or a more than a half of the spore cavity. Polar capsules of equal sizes. Suture line well expressed; sometimes slightly folded. Spore dimensions from glycerine jelly mounts were 6.19 ± 0.29 μm (5.56–6.75) in length; 7.22 ± 0.28 μm (6.57–7.77) in width, and 4.60 ± 0.36 μm (3.55–5.27) in thickness (*n* = 50). Polar capsules measured 3.07 ± 0.32 μm (2.36–3.8) × 1.81 ± 0.22 μm (1.26–2.28). Four coiled polar filament measured 13.45 ± 1.95 μm (12.0–17.76) in length.

*Syntype specimens*. Glass slides numbers AAK 7, 15, 19, 20, 21, 22, 23, 29, 33, 37, 44; AAG 6, 8, 13, 38, 42, 51, 63, 64, 136, 147, 148; MAE 31; 2 MAE 21, 26, 39, 56, 65; 2 MAS 3, 4, 5, 6, 7, 8, 11, 12; 3 MAE 17, 20, 49; 3 MAS 4, 7, 8, 13, 17, 32, 35; and 4 MAE 9, 10, 12, 18, 23, 24, 29, 31 were deposited in the collection of the Department of Parasitology of Institute of Biology of the Southern Seas of National Academy of Sciences of Ukraine, 2 Nakhimov Avenue, 99011, Sevastopol, Ukraine

*Etymology*. Species is called to the honor of Adel Kovalyova, expert on Myxosporea studies, who worked longtime in the Institute of Biology of the Southern Seas (IBSS) and Fish Diseases Laboratory AtlantNIRO, Kaliningrad, Russia

*Taxonomic summary.* The new myxosoporean species differs from other representatives of the genus *Myxobolus* by morphology and spore sizes. The spore shape and/or measurements of the present species showed some similarities with Myxosporea from the Eurasia freshwater hosts: *M. improvisus* Isjumova, 1964 in. Schulman 1966; *Myxobolus latus* Schulman, 1962 and *Myxobolus artus* Achmerov, 1960. *M. adeli* sp. produces spindle-shaped plasmodia contrary to *M. improvisus* and *M. latus* with round- or oval-shaped (*M. artus*) vegetative stages. The spores of newly described species are comparatively smaller than the spores of all three related species. *M. adeli* sp. n. differs from *M. improvisus* also by equal sized polar capsules (Table 2).

*Alataspora* sp. (Table 3; Figs. 16, 17)

**Table 3:** *Alataspora* sp. measurements

Plasmodia and spores measurements	Fresh material (*n* = 20)	Smears colored with Giemsa stain (*n* = 22)
Plasmodium length	15.65 ± 5.58 (5.5–26.5)	15.02 ± 6.38 (4.01–34.38)
Plasmodium width	14.7 ± 4.89 (5.5–23.5)	13.78 ± 5.5 (3.33–25.03)
Spore length	8.3 ± 0.54 (7.5–9.0)	9.9 ± 1.08 (8.10–11.56)
Spore thickness	24.16 ± 3.0 (19.0–28.5)	24.29 ± 3.22 (19.76–29.85)
Thickness of bigger valve	13.8 ± 1.58 (12.0–17.0)	12.8 (10.32–15.91)
Thickness of smaller valve	11.67 ± 1.37 (8.5–13.0)	11.61 (9.44–13.94)
Polar capsule length	3.1 ± 0.08 (3.0–3.3)	2.77 ± 0.32 (2.2–3.21)
Polar capsule width	3.1 ± 0.08 (3.0–3.3)	2.52 ± 0.34 (1.78–3.11)
Number of polar filament coils	5	–

*Type host*. Thinlip mullet *L. ramada* (Risso, 1826)

*Site of infection*. Gall bladder

*Locality.* Mediterranean coastal waters (Ebro River Delta, Santa Pola)

*Prevalence*. 2.7 % (1/37) in 2004; 0.9 % (1/109) in 2005

*Description.* Vegetative stages presented by rounded or oval-shaped bisporous plasmodia with transparent ectoplasm and small-grained endoplasm. Spores are strongly elongated in the plane perpendicular to the sutural line. They have clearly expressed triangular part, cavity of which contains polar capsules and amoeboid germ. Elongated top parts of the valves form single wing-like appendages slightly unequal in sizes. Suture line is straight and clear. Spherical polar capsules are located close to the anterior pole and open near the suture line to one side of spore. Amoeboid germ is located under polar capsules.

Spore measurements presented in Table 3.

*Taxonomic summary.* Based on the spore construction, *Alataspora* sp. occupies intermediate position between representatives of *Alataspora* and *Pseudalataspora* genera. It resembles *Alataspora solomoni* Yurakhno, 1988, differing from it by unequal length of valves and larger spores and polar capsules. We consider *Alataspora* sp. a *species inquirenda* that needs a precise species description after obtaining of additional data.

## References

[CR1] Asejeva NL (1994). Detection on *Myxosoma acutum* in pilengas from Japan Sea. Izvestiya TINRO.

[CR2] Asejeva NL (2000). Myxosporeans of anadrome and marine coastal fishes of north-west part of Japan Sea. Izvestiya TINRO.

[CR3] Bahri S, Marques A (1996). Myxosporean parasites of the genus *Myxobolus* from *Mugil cephalus* in Ichkeul lagoon, Tunisia: description of two new species. Dis Aquat Org.

[CR4] Bahri S, Andree KB, Hedrick RP (2003). Morphological and phylogenetic studies of marine *Myxobolus* spp. from mullet in Ichkeul Lake, Tunisia. J Eukaryot Microbiol.

[CR5] Bykhovskaya-Pavlovskaya IE (1985). Parasites of fish: guide for the study.

[CR6] Chen CL, Hsieh SR (1984) New species of *Myxidium* (Myxosporidia) from freshwater fishes of China. In: Parasitic organisms of freshwater fish of China. Institute of Hydrobiology, Academia Sinica, Agricultural Publishing House, Beijing pp 89–8 (in Chinese with English abstract)

[CR7] Diamanka A, Fall M, Diebakate C, Faye N, Toguebaye BS (2008). Identification of *Myxobolus episquamalis* (Myxozoa, Myxobolidae) in flathead mullet *Mugil cephalus* (Pisces, Teleostei, Mugilidae) from the coast of Senegal (eastern tropical Atlantic Ocean). Acta Adriat.

[CR8] Diamant A, Ucko M, Paperna I, Colorni A, Lipshitz A (2005). *Kudoa iwatai* (Myxosporea: Multivalvulida) in wild and cultured fish in the Red Sea: redescription and molecular phylogeny. J Parasitol.

[CR9] Domnich IF, Sarabeev VL (1999). Parasitic fauna of Azov Sea grey mullet and the ways of its formation. Visnyk Zaporizkogo Derzhavnogo Universytetu (Vistnyk Zaporizhzhya National Univ).

[CR10] Domnich IF, Sarabeev VL (2000). The present fauna of fish parasites in the northern part of Azov Sea. Visnyk Zaporizkogo Derzhavnogo Universytetu (Vistnyk Zaporizhzhya National Univ).

[CR11] Donets ZS (1979) The zoogeographical analysis of Myxosporidia in the USSR southern water reservoirs. In: Evolution and ecology of Sporozoa and Cnidosporidia. Tr Zool Inst Akad Nauk SSSR 87:65–90 (In Russian)

[CR12] Donets ZS, Schulman SS (1973). About methods of Myxosporidia (Protozoa, Cnidosporidia) investigation. Parazitologiya.

[CR13] Dorothy KP, Kalavati C (1992). Two new myxosporean parasites of the mullet *Liza macrolepis* (Smith). Uttar Pradesh J Zool.

[CR14] Egusa S, Maeno Y, Sorimachi M (1990). A new species of Myxozoa, *Myxobolus episquamalis* sp. n. infecting the scales of the mullet, *Mugil cephalus* L. Fish Pathol.

[CR15] Eiras JC (2002). Synopsis of the species of the genus *Henneguya* Thelohan, 1892 (Myxozoa: Myxosporea: Myxobolidae). Syst Parasitol.

[CR16] Eiras JC, D’Souza J (2004). *Myxobolus goensis* n. sp. (Myxozoa, Myxosporea, Myxobolidae), a parasite of the gills of *Mugil cephalus* (Osteichthyes, Mugilidae) from Goa, India. Parasite.

[CR17] Eiras JC, Molnar K, Lu YS (2005). Synopsis of the species of *Myxobolus* Butschli, 1882 (Myxozoa: Myxosporea: Myxobolidae). Syst Parasitol.

[CR18] Eiras JC, Abreu PC, Robaldo R, Pereira Junior J (2007). *Myxobolus platanus* n. sp. (Myxosporea, Myxobolidae), a parasite of *Mugil platanus* Gunther, 1880 (Osteichthyes, Mugilidae) from Lagoa dos Patos, RS, Brasil. Arq Bras Med Vet Zootec.

[CR19] Ergens R, Gussev AV, Izyumova NA, Molnar K (1975) Parasite fauna of fishes of the Tisa River Basin. Praha 117 p

[CR20] Fall M, Kpatcha KP, Diebakate C, Faye N, Toguebaye BS (1997). Observations sur des Myxosporidies (Myxozoa) du genre *Myxobolus parasites* de *Mugil cephalus* (Poisson, Téléostéen) du Sénégal. Parasite.

[CR21] Faye N, Kpatcha K, Fall M, Toguebaye BS (1997). Heart infections due to myxosporean (Myxozoa) parasites in marine and estuarine fishes from Senegal. Bull Eur Assoc Fish Pathol.

[CR22] Fujita T (1930). On the new Myxosporidia *Chloromyxum bora* nov. sp. In the muscles of the Gray-Mullet. Dibitsugaku zasshi Tokyo.

[CR23] Haldar DP, Samal KK, Mukhopadhyaya D (1996). Studies on the protozoan parasites of fishes in Orissa: eight species of *Myxobolus* Butschli (Myxozoa: Bivalvulida). J Bengal Nat History Soc.

[CR24] Holzer AS, Blasco-Costa I, Sarabeev VL, Ovcharenko MO, Balbuena JA (2006). *Kudoa trifolia* sp. n.—molecular phylogeny suggests a new spore morphology and unusual tissue location for a well-known genus. J Fish Dis.

[CR25] Ibragimov SR (1987) Forming of the parasites fauna of mullets in the Caspian Sea. Baku, Institute of Zoology of AS Aserbaijan SSR (Deposited by VINITI 03.04.87, № 2407-B87):1–14 (In Russian)

[CR26] Iskov MP (1989) Myxosporidia (Myxosporea). In: Markevitch AP, Schulman SS (eds) Fauna Ukrainy, vol. 37 (4), Naukowa Dumka, Kiev: 212 p. (In Russian)

[CR27] Iversen ES, Chitty N, van Meter N (1971). Some myxosporida from marine fishes in south Florida. J Protozool.

[CR28] Jajasri M, Hoffman GL (1982). Review of *Myxidium* (Protozoa: Myxozoa: Myxosporea). Protozool Abstr.

[CR29] Kalavati C, Anuradha I (1993). Two new species of myxosporeans infecting *Valamugil cunnesius* in Visakhaptnam harbour, east coast of India. Uttar Pradesh J Zool.

[CR30] Kalavati C, Anuradha I (1995). A new myxosporean, *Bipteria indica* sp. n. (Myxospora: Sinuolineidae) from the gall bladder of the striped mullet, *Mugil cephalus* L. Acta Protozool.

[CR31] Karatajev AK, Iskov MP (1984). The materials on the fauna of protozoa—fish parasites in the Black Sea north-western part. Vestn Zool.

[CR32] Kent ML, Andree KB, Bartholomew JL, El-Matbouli M, Desser SS, Delvin RH, Feist SW, Hedrick RP, Hoffmann RW, Khattra J, Hallett SL, Lester RJG, Longshaw M, Palenzuela O, Siddall ME, Xizo CX (2001). Recent advances in our knowledge of the Myxozoa. J Eukaryot Microbiol.

[CR33] Kim WS, Kim JH, Jang MS, Jang SJ, Oh MJ (2013). Infection of wild mullet (*Mugil cephalus*) with *Myxobolus episquamalis* in Korea. Parasitol Res.

[CR34] Kolesnikova MG, Donets ZS (1987) The fauna of fish Myxosporidia at the Crimean coast. IV-th All Union Symposium “Parasitology and Pathology of marine organisms” (21–23 April, 1987, Kaliningrad): Abstracts:89–90 (In Russian)

[CR35] Kovaleva AA, Donets ZS, Kolesnikova MG (1989). New species of Myxosporidia (Cnidospora, Myxosporea) in the Black Sea fish. Vestn Zool.

[CR36] Kpatcha TK, Faye N, Diebakate C, Fall M, Toguebaye BS (1997). Nouvelles espèces d.*Henneguya* Thelohan, 1895 (Myxozoa, Myxosporea) parasites des poissons marins du Sénégal: étude en microscopie photonique et électronique. Ann Sci Nat Zool Paris.

[CR37] Kudo R (1919). A synopsis on genera and species of Myxosporidia. Illinois Biol Monogr.

[CR38] Landsberg JH, Lom J (1991). Taxonomy of the genera of the *Myxosoma/Myxobolus* group (Myxobolidae: Myxosporea), current listing of species and revision of synonyms. Syst Parasitol.

[CR39] Lom J, Arthur JR (2006). A guideline for the preparation of species descriptions in Myxosporea. J Fish Dis.

[CR40] Lom J, Dyková I (1992). Protozoan parasites of fishes. Developments in aquaculture and fisheries sciences.

[CR41] Lom J, Dyková I (1994). Studies on protozoan parasites of Australian fishes III. Species of the genus *Myxobolus* Bűtschli, 1882. Eur J Protistol.

[CR42] Lubat V, Radujkovic B, Marques A, Bouix G (1989). Parasites des poissons marins du Montenegro: Myxosporidies. Acta Adriat.

[CR43] Maeno Y, Sorimachi M, Ogawa K, Egusa S (1990). *Myxobolus spinacurvatura* sp. n. (Myxosporea: Bivalvulida) parasitic in deformed mullet, *Mugil cephalus*. Fish Pathol.

[CR44] Maeno Y, Nagasawa K, Sorimachi M (1993). *Kudoa intestinalis* n. sp. (Myxosporea: Multivalvulida) from the intestinal musculature of the striped mullet, *Mugil cephalus*, from Japan. J Parasitol.

[CR45] Moran JDW, Whitaker DJ, Kent ML (1999). A review of the myxosporean genus *Kudoa* Meglitsch, 1947, and its impact on the international aquaculture industry and commercial fisheries. Aquaculture.

[CR46] Munoz P, Palenzuela O, Alvarez-Pelitero P, Sitja-Bobadilla A (1999). Comparative studies on carbohydrates of several myxosporean parasites of fish using lectin histochemical methods. Folia Parasitol.

[CR47] Naidenova NN, Schulman SS, Donets ZS (1975) Type protozoa, class Myxosporidia. In: Guide of parasites of vertebrates of Black and Azov Seas Naukova Dumka Kiev:20–50 (In Russian)

[CR48] Narasimhamurti CC (1970). *Myxosoma intestinalis* n. sp. (Protozoa, Myxosporidia) parasiting in the intestinal epithelium in the estuaria fish *Mugil waigensis*. Proc Indian Acad Sci.

[CR49] Narasimhamurti CC, Kalavati C (1979). *Myxosoma lairdi* n. sp. (Protozoa: Myxosporidia) parasitic in the gut of the estuarine fish *Liza macrolepis* Smith. Proc Indian Acad Sci.

[CR50] Narasimhamurti CC, Kalavati C (1979). *Kudoa tetraspora* n. sp. (Protozoa: Myxosporidea: Protozoa) parasitic in the brain tissue of *Mugil cephalus*. Proc Indian Acad Sci.

[CR51] Narasimhamurti С, Kalavati С, Saratchandra B (1980). *Myxosoma microspora* n. sp. (Myxosporidia: Protozoa) parasitic in the gills of *Mugil cephalus*. J Fish Biol.

[CR52] Negm-Eldim NM, Govedich FR, Davies RW (1999). Gill myxosporeans on some Egyptian freshwater fish. Deutsche Tierarzt Wochenschr.

[CR53] Nelson JS (1984). Fishes of the world.

[CR54] Özak AA, Demirkale I, Cengizler I (2012). Two new records of *Myxobolus* Butschli, 1882 (Myxozoa, Myxosporea, Myxobolidae) species from Turkey. Turk J Zool.

[CR55] Padros F, Palenzuela O, Hispano C, Tosas O, Zarza C, Crespo S, Alvarez-Pellitero P (2001). *Myxidium leei* (Myxozoa) infections in aquarium-reared Mediterranean fish species. Dis Aquat Org.

[CR56] Paperna I (1975). Parasites and disease of the grey mullet (Mugilidae) with special reference to the seas of the Near East. Aquaculture.

[CR57] Paperna I, Overstreet RM, Oren OH (1981). Parasites and diseases of mullets (Mugilidae). Aquaculture of grey mullets. IBP 26.

[CR58] Parenzan P (1966). *Myxobolus mugilis* e *Myxobolus branchialis* nuovi missosporidi parassiti di *Mugil chelo* dello Jonio. Bull Soc Nat Napoli.

[CR59] Parisi B (1912). Primo contributo alla distribuzione geographica dei Missosporidi in Italia (Milano). Atti Soc Ital Sci Nat.

[CR60] Pedro-Andrĕs MB, Marques A, Gracia-Royo MP (2011). Myxosporean infection of grey mullet in the Ebro Delta: identification and ultrastructure of *Myxobolus ichkeulensis* Bahri & Marques, 1996 infecting the gills of *Mugil cephalus* L. Acta Protozool.

[CR61] Perugia A (1891). Sulle Missosporidie dei Pesci marini. I. Boll Sci.

[CR62] Pogoreltceva TP (1952). The materials on the fish parasite fauna of the Black Sea southern-eastern part. Tr Inst Zool Akad Nauk Ukr SSR.

[CR63] Pogoreltceva TP (1964). Materials on investigations of parasitic Protozoa of Black Sea fishes. Problemy Parazitologii. Tr Ukr Resp Nauchn O-va Parazitol.

[CR64] Pulsford A, Matthews RA (1982). An ultrastructural study on *Myxobolus exiguus* Thelohan, 1895 (Myxosporea) from grey mullet *Crenimugil labrosus* (Risso). J Fish Dis.

[CR65] Quaglio F, Delgado ML, Caffara M, Florio D, Marcer F, Fioravanti ML, Restani R (2002). Histopathological observations in marine farmed fish infected by Myxosporidia. II Osservazioni istopatologiche in pesci marini d’allevamento affetti da mixosporidiosi. II. Boll Soc Ital Patol Ittica.

[CR66] Reshetnikova AV (1955). Parasitic fauna of the Black sea mullets. Tr Karadag Biol Stn Akad Nauk Ukr SSR.

[CR67] Rothwell JT, Virgona JL, Callinan RB, Nicholls PJ, Langdon JS (1997). Occurrence of cutaneous infections of *Myxobolus episquamalis* (Myxozoa: Myxobolidae) in sea mullet, *Mugil cephalus* L. in Australia. Aust Vet J.

[CR68] Sarabeev VL, Domnich IF (2000). The age dynamics in haarder *Mugil soiuy* infection in the sea of Azov Molochny gulf. Vestn Zool.

[CR69] Sarkar NK (1989). *Myxobolus anili* sp. nov. (Myxozoa: Myxosporea) from a marine teleost fish *Rhinomugil corsula* Hamilton. Proc Zool Soc Calcutta.

[CR70] Sarkar NK (1999). Some new Myxosporidia (Myxozoa: Myxosporea) of the genera *Myxobolus* Butschli, 1882 *Unicapsula* Davis, 1942 *Kudoa* Meglitsch, 1947 *Ortholinea* Shulman, 1962 and *Neoparvicapsula* Gajevskaya, Kovaleva and Shulman, 1982. Proc Zool Soc Calcutta.

[CR71] Sarkar NK, Chaudhury SR (1996). *Kudoa cascasia* sp. n. (Myxosporea: Kudoidae) parasitic in the mesentery of *Sicamugil cascasia* (Ham.) from Hoodghly estuary of West Bengal, India. Acta Protozool.

[CR72] Sarkar NK, Ghosh S (1991). Two new coelozoic myxosporida (Myxozoa: Myxosporea) from estuarine teleost fishes (Mugilidae) of West Bengal, India. Proc Zool Soc Calcutta.

[CR73] Schulman SS (1957). About pathogenicity of myxosporea *Myxobolus exiguus* and it-associated epizooties. Izv Vses Nauchno-Issled Inst Ozern Rechn Rybn Khoz.

[CR74] Schulman SS (1962) Class Cnidosporidia. In: Opredelitel parasitov presnovodnykh ryb SSSR AN SSSR, Moscow :47–130 (In Russian)

[CR75] Schulman SS (1966). Myxosporidia of the fauna of the USSR.

[CR76] Schulman SS, Donets ZS, Kovaleva AA (1997). Class of myxosporeans (Myxosporea) of the world fauna. Vol 1.

[CR77] Siau Y (1978) Contribution á la coinnaissance des Microsporidies: etude de *Myxobolus exiguous* Thélohan, 1895 (cytology, cycle, actions sur l’hôte, epidemiologie). Thesis USTL (Université Sciences et Techniques du Lanquedoc), Montpellier II

[CR78] Sitjà-Bobadilla A, Alvarez-Pellitero P (1993). *Zschokkella mugilis* n. sp. (Myxosporea: Bivalvulida) from mullets (Teleostei: Mugilidae) of Mediterranean waters: light and electron microscopic description. J Eukaryot Microbiol.

[CR79] Sitjà-Bobadilla A, Alvarez-Pellitero P (1994). Revised classification and key species of the genus *Sphaerospora* Davies, 1917 (Protozoa: Myxosporea). Res Rev Parasitol.

[CR80] Sitjà-Bobadilla A, Alvarez-Pellitero P (1995). Light and electron microscopic description of *Polysporoplasma* n. g. (Myxosporea: Bivalvulida), *Polysporoplasma sparis* n. sp. from *Sparus aurata* (L.), and *Polysporoplasma mugilis* n. sp. from *Liza aurata* L. Eur J Protistol.

[CR81] Sitjà-Bobadilla A, Alvarez-Pellitero P (1996). Virus-like particles in *Polysporoplasma mugilis* (Protozoa: Myxosporea), parasitic in a marine fish (*Liza aurata* L.). Int J Parasitol.

[CR82] Syirovatka NI, Nizova GA (2000) Formation of haarder parasitic fauna in the Azov basin water reservoirs. In: Trudy AzNIIRH (1998–1999). The main problems of fish economics and protection of fish farms water reservoirs in the Azov-Black sea basin, (Ed. Makarov), BKI, Rostov-on-Don: 172–176 (In Russian)

[CR83] Thélohan P (1895). Recherches sur les Myxosporidies. Bull Sci Fr Belg.

[CR84] U-Taynapun K, Penprapai N, Bangrak P, Mekata T, Itami T, Tantikitti C (2011). *Myxobolus supamattayai* n. sp. (Myxosporea: Myxobolidae) from Thailand parasitizing the scale pellicle of wild mullet (*Valamugil seheli*). Parasitol Res.

[CR85] Vávra J, Maddox JV, Bulla LA, Cheng TC (1976). Methods in microsporidiology. Comparative pathobiology.

[CR86] Yemmen C, Ktari MH, Bahri S (2012). Parasitofauna of some mugilid and soleid fish species from Tunisian lagoons. Acta Adriat.

[CR87] Yurakhno VM (1993). New data of the fauna of myxosporidians from fishes of the Black Sea. Parazitologiya.

[CR88] Yurakhno VM, Nigmatullin CM (2004). The fauna of myxosporeans (Protozoa: Myxosporea) of fishes in the Black Sea and its seasonal and interannual aspects of variability. Sovremennyje problemy parazitologii, zoologii i ekologii.

[CR89] Yurakhno VM, Maltsev VN (2002). New data on myxosporeans of mullets in the Atlantic Ocean basin. Ekologija Morya.

[CR90] Yurakhno VM, Ovcharenko M (2008) Myxosporeans of the world ocean mullets. Proceedings of the IV Congress of the Russian Society of Parasitologists – Russian Academy of Sciences, held 20–25 October 2008 at the Zoological Institute RAS, St. Petersburg, “Parasitology in XXI century – problems, methods, solutions”, Vol. 3, St. Petersburg: 231–234

[CR91] Yurakhno VM, Ovcharenko MO, Holzer AS, Sarabeev VL, Balbuena JA (2007). *Kudoa unicapsula* n. sp. (Myxosporea: Kudoidae) a parasite of the Mediterranean mullets *Liza ramada* and *L. aurata* (Teleostei: Mugilidae). Parasitol Res.

